# Fasting plasma methylglyoxal concentrations are associated with higher numbers of circulating intermediate and non-classical monocytes but with lower activation of intermediate monocytes: the Maastricht Study

**DOI:** 10.1007/s40618-025-02536-1

**Published:** 2025-01-23

**Authors:** Xiaodi Zhang, Marleen M. J. van Greevenbroek, Jean L. J. M. Scheijen, Simone J. P. M. Eussen, Jaycey Kelly, Coen D. A. Stehouwer, Casper G. Schalkwijk, Kristiaan Wouters

**Affiliations:** 1 Department of Internal Medicine, Maastricht University Medical Center, Maastricht, 6229ER the Netherlands; 2 CARIM School for Cardiovascular Diseases, Maastricht University, Maastricht, 6229ER the Netherlands; 3 Department of Epidemiology, Maastricht University, Maastricht, 6229HA the Netherlands; 4 CAPHRI School for Care and Public Health Research Unit, Maastricht University, Maastricht, 6229ER the Netherlands

**Keywords:** Methylglyoxal, Circulating monocytes, Neutrophils, Population-based cohort study, Type 2 diabetes, Cardiovascular disease

## Abstract

**Purpose:**

Elevated methylglyoxal (MGO) levels and altered immune cell responses are observed in diabetes. MGO is thought to modulate immune cell activation. The current study investigated whether fasting or post-glucose-load plasma MGO concentrations are associated with circulating immune cell counts and activation in a large cohort study.

**Methods:**

696 participants of The Maastricht Study (age 60.3 ± 8.4 years, 51.9% women) underwent an oral glucose tolerance test (OGTT). Fasting and post-OGTT plasma MGO concentrations were measured using mass spectrometry. Numbers and activation of circulating immune cells at fasting state were quantified using flow cytometry. Activation scores were calculated by averaging individual marker z-scores for neutrophils (CD11b, CD11c, CD16) and classical, intermediate, and non-classical monocytes (CD11b, CD11c, CX3XR1, HLA-DR). Associations were analysed using multiple linear regression adjusted for potential confounders. Stratified analyses were performed for glucose metabolism status for associations between plasma MGO levels and immune cell counts.

**Results:**

Higher fasting plasma MGO concentrations were significantly associated with higher numbers of intermediate (β = 0.09 [95%CI 0.02; 0.17]) and non-classical monocytes (0.08 [0.002; 0.15]), but with lower activation scores for the intermediate monocytes (-0.14 [-0.22; -0.06]). Stratified analyses showed that positive associations between fasting plasma MGO levels and numbers of intermediate and non-classical monocytes appear only in participants with type 2 diabetes. Post-OGTT plasma MGO concentrations were not consistently associated with immune cells counts or activation.

**Conclusion:**

Higher fasting plasma MGO concentrations are associated with higher intermediate and non-classical monocyte counts but with lower activation of intermediate monocytes.

**Supplementary Information:**

The online version contains supplementary material available at 10.1007/s40618-025-02536-1.

## Introduction

Increased formation of methylglyoxal (MGO), a highly reactive dicarbonyl compound, is a potential driver of hyperglycaemia-induced vascular complications and cardiovascular disease (CVD) in diabetes [1–6]. Endogenous MGO formation is mainly derived from the non-enzymatic degradation of the trioses glyceraldehyde 3-phosphate and dihydroxyacetone phosphate, which are formed during glycolysis [1]. We previously showed that plasma MGO levels are increased in the postprandially phase with higher concentrations in individuals with impaired glucose metabolism and type 2 diabetes [7, 8]. Both fasting and post-oral-glucose-load levels of MGO are involved in the development of hyperglycaemia-related vascular complications [2, 3, 9], but the underlying mechanism is unknown.

Immune cells like monocytes and neutrophils are particularly sensitive to hyperglycaemia and contribute to glucose-induced inflammation [4, 10]. Both activation and numbers of monocytes and neutrophils have been closely associated with type 2 diabetes (T2D) and contribute to the development of its complications, especially atherosclerosis [10–14]. Hyperglycaemic spikes in mice led to increased myelopoiesis in bone marrow as well as circulating neutrophils and monocytes [10]. Furthermore, under hyperglycaemic condition, neutrophils are damaged as indicated by increased expression of CD11b and its ligand intercellular adhesion molecule-1 (ICAM-1). As a key adhesion receptor, CD11b can bind to ICAM-1 and prompt neutrophils to adhere to endothelial cells and subsequently migrate across endothelial cells to the inflammatory spot [15].

Monocytes are heterogeneous, and shifts in monocyte subset distribution have been reported in the context of CVD [16]. Based on the differences in expression of the lipopolysaccharide receptor CD14 and the Fc gamma receptor CD16, human circulating monocytes are subdivided into classical (CD14^+^CD16^−^), intermediate (CD14^+^CD16^+^), and non-classical (CD14^dim^CD16^+^) monocytes [17, 18]. Classical monocytes are highly active in phagocytosis in response to pathogens and they differentiate into nonclassical monocytes at steady state [19]. Non-classical monocytes are believed to patrol the vascular endothelium in homeostasis and play a role in the maintenance of vascular integrity [16]. The function of intermediate monocytes remains less clear, however, they appear to be the most predictive of CVD, and they have been shown to be associated with vulnerable atherosclerotic plaque, acute myocardial infarction, and heart failure [16].

It has been suggested that MGO may contribute to the induction of immune cell activation [20]. Chronic exposure to MGO led to an activated neutrophil phenotype [20, 21]. Ex vivo MGO treatment induced monocyte migration and adhesion to endothelial cells [20, 22, 23]. However, the association between in vivo MGO levels and immune cell responses remains unclear. Since MGO and immune cells are both linked with diabetes and its complications, we hypothesized that changes in plasma MGO levels are associated with circulating immune cell responses. In the current study we investigated the associations of plasma MGO levels prior to and after an oral glucose tolerance test (OGTT) with circulating immune cell counts and activation, in subjects with normal glucose metabolism (NGM), prediabetes, and type 2 diabetes (T2D).

## Patients and methods

### Study design and population

The current study used data from the Maastricht Study, an observational prospective population-based cohort study with an oversampling of individuals with type 2 diabetes. The rationale and methodology have been extensively described previously [24]. In brief, the study focuses on the aetiology, pathophysiology, complications, and comorbid conditions of type 2 diabetes and is characterized by an extensive phenotyping approach. Eligible for participation were all individuals aged 40 to 75 years and living in the southern part of the Netherlands. Participants were recruited through mass media campaigns and from the municipal registries and the regional Diabetes Patient Registry by mailings. The present study includes a subset of the cross-sectional data from the first 7689 participants, who completed the baseline survey between November 2010 and December 2017. The examinations of each participant were performed within a time window of 3 months.

The study has been approved by the institutional medical ethics committee (NL31329.068.10) and the Dutch Ministry of Health, Welfare, and Sports of the Netherlands (permit 131088-105234-PG). All participants gave written informed consent.

## Oral glucose tolerance test

All participants underwent a standard 75 g OGTT (2 h) after an overnight fast, except for the ones who use insulin or with a fasting glucose level above 11.0 mmol/L (as determined by finger-prick test) for safety reasons. Blood samples were collected in ethylenediaminetetraacetic acid (EDTA) tubes at baseline and at time point 120 min of the OGTT. Blood tubes were placed on ice immediately and were used within 3 h after collection. Plasma samples were obtained by spinning the blood for 10 min at 2000 g, 4 °C, and were stored at −80 °C until analysis. Glucose metabolism status was defined according to the WHO 2006 criteria as normal glucose metabolism (NGM), prediabetes (impaired fasting glucose or impaired glucose tolerance), and type 2 diabetes (T2D) [25].

## Measurements of plasma methylglyoxal

Concentrations of plasma MGO were measured by ultra-performance liquid chromatography tandem mass spectrometry (UPLC-MS/MS), as described previously [26]. Briefly, 30 µL EDTA plasma was mixed with 90 µL O-phenylenediamine (oPD, 10 mg oPD in 10 mL 1.6 mol/L perchloric acid). After an overnight (20 h) reaction at room temperature and shielded from light, 20 µL of d4-MGO-oPD internal standard solution was added. Samples were mixed and then centrifuged at 21,000 g, 4 °C, for 20 min. 5 µL was injected for UPLC-MS/MS analysis.

## Measurements of immune cell subsets

Blood samples were collected in heparin tubes from fasting participants at the first visit and the total leukocyte count was quantified by the automated blood cell count (Sysmex XE5000, Kobe, Japan). Additionally, 50 µL of fresh heparinized whole blood was used for flow cytometry analysis. The staining cocktail included the following antibodies: CD3-FITC (BD 561807), CD19-FITC (BD 555412), CD66b-FITC (BD 555724), CD56-PE (BD, 345810), HLA-DR-V500 (BD 561224), CD14-APC-H7 (BD 641394), CD16-PerCP (BD 560717), CD11b-BV421 (BD 562632), CD11c-PE-Cy7 (BD 561356), and CX3CR1-APC (Biolegend 341609). From single blood cells, live cells were gated based on forward and side scatters. Neutrophils (CD66b^+^) and monocytes (CD3^−^CD19^−^CD66b^−^HLA-DR^+^) were identified from live cells, monocyte subsets (classical monocytes: CD14^+^CD16^−^, intermediate monocytes: CD14^+^CD16^+^, nonclassical monocytes: CD14^dim^CD16^+^) were gated out of the monocyte population, as described previously [17]. The CD56 marker was used in the staining cocktail for identification of natural killer cells and not used in the current study.

## Calculations of immune cell counts

The flow cytometry-based total events of the monocytes were calculated as the sum of events from the classical, intermediate, and non-classical monocytes. The percentages of monocytes and neutrophils were calculated from the live cell gate, and percentages of monocyte subsets were calculated from the parent monocyte gate. These percentages were then used to calculate the actual cell counts (x 10^9^ per liter blood) based on the total leukocyte count measured by the automated blood cell counter.

### Calculations of immune cell activation score

CD11b, CD11c, and CD16 were used as neutrophil activation markers [27, 28], and CD11b, CD11c, CX3CR1, and HLA-DR were used as monocyte activation markers [17, 29]. Sum scores of the neutrophil and monocyte activation markers were calculated according to a predefined cluster of conceptually related biomarkers [30]: for each individual marker, mean fluorescence intensities (MFIs) were ln-transformed and substantially a z-score was calculated using the total sample’s mean and standard deviation. The resulting individual marker z-scores were averaged and standardized again to yield an overall standardized sum score for neutrophil and monocyte activation, respectively.

## Covariates

Education status, smoking status, and medication use were assessed by a questionnaire or an interview. Education status was categorized into low, medium, and high. Smoking status was categorized into never, former, and current smoker. Body mass index (BMI), waist circumference, and systolic blood pressure (SBP) were measured by physical examination, as described elsewhere [24]. Physical activity was assessed using the Community Health Activities Model Program for Seniors physical activity self-report questionnaire [31]. The Dutch Healthy Diet (DHD) index, which reflects adherence to the Dutch dietary guidelines, was based on a food frequency questionnaire, as described previously [32].

## Statistical analyses

All statistical analyses were performed with SPSS version 25.0 for Windows (IBM Corporation, Armonk, NY, USA). Baseline characteristics are shown for the total study population and stratified according to glucose metabolism status. Differences in characteristics between participants with different glucose metabolism status were analysed by means of a One-way ANOVA or chi-squared test, for comparisons of continuous or categorical variables, respectively.

Multiple linear regression was used to investigate cross-sectional associations of plasma MGO levels with immune cell counts and immune cell activation scores. Plasma MGO levels and immune cell counts were ln-transformed to ensure normality and then standardized, prior to analyses. Five regression models were fitted. Model 1 was the crude model; model 2 was adjusted for age (years) and sex (male/female); model 3 was additionally adjusted for potential confounders related to lifestyle: BMI (kg/m^2^), education status (low, medium, high), and smoking status (never, former, current); model 4 was additionally adjusted for systolic blood pressure (mmHg), and use of medications: glucose-lowering, anti-hypertensive, and lipid-lowering medication (each yes/no); model 5 was additionally adjusted for glucose metabolism status (NGM, prediabetes, T2D) to account for oversampling of individuals with T2D in The Maastricht Study.

Interaction analyses were performed for sex (sex × MGO, sex × each individual covariate), and glucose metabolism status (prediabetes or T2D × MGO, prediabetes or T2D × each individual covariate) by adding interaction terms to model 5. *p*_interaction_ < 0.05 was considered statistically significant for these analyses.

To test the robustness of our results, sensitivity analyses were conducted, replacing BMI by waist circumferences in model 3, or adding DHD score and physical activity to the fully adjusted model. The results are presented as the beta regression coefficient and the corresponding 95% confidence intervals (CI). A *p* value of < 0.05 was considered statistically significant.

## Results

### Population characteristics

**Fig. 1:**
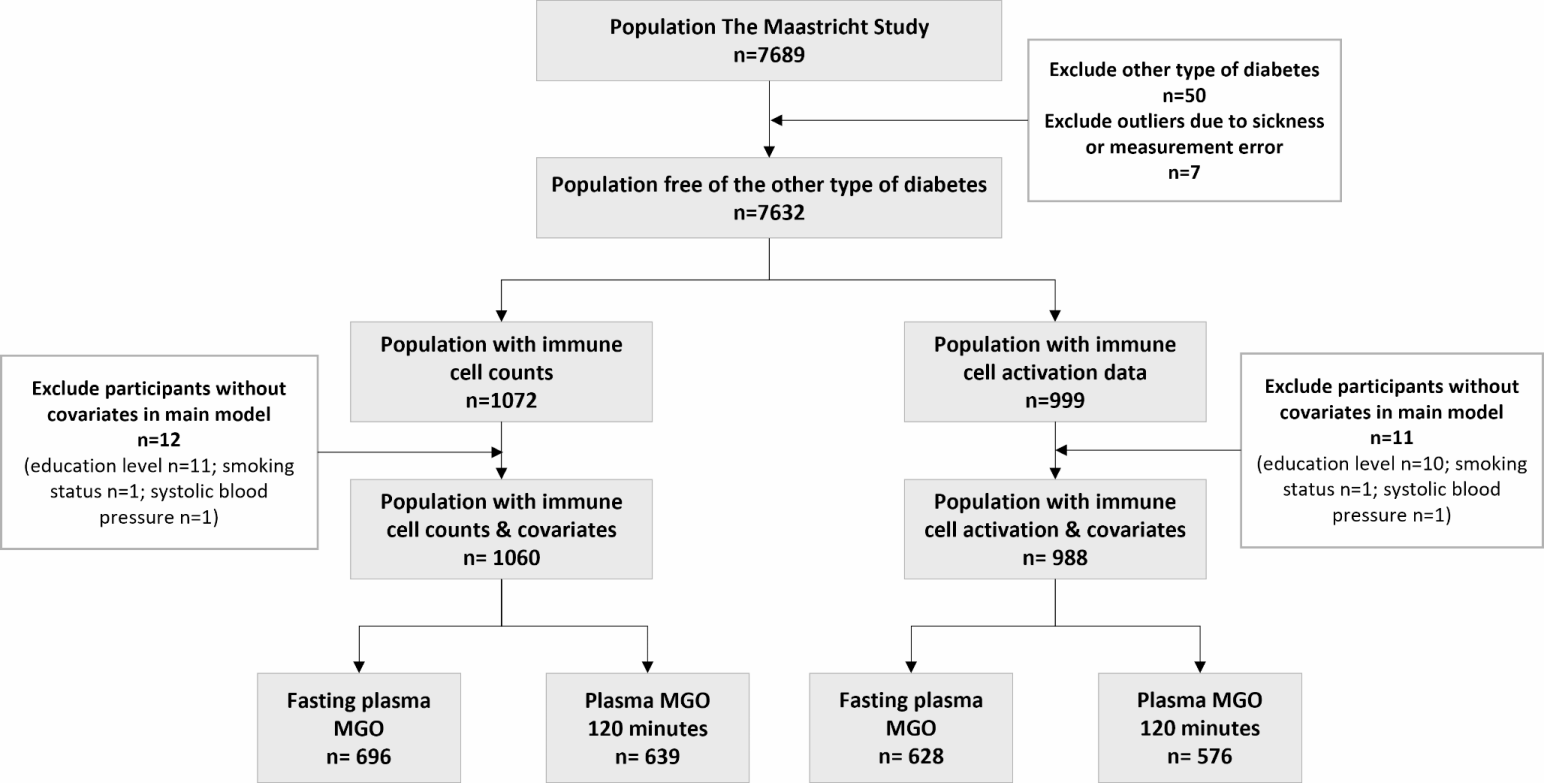
Flowchart of the study population selection process

The characteristics of participants with available data on plasma MGO concentrations, immune cell counts and immune cell activation markers, and potential confounders are shown in Fig. 1. Characteristics of the study population, according to glucose metabolism status are shown in Table 1. The participants with T2D were generally older and had a higher BMI, waist circumference, HbA1c levels and systolic blood pressure, compared with the groups without diabetes. Additionally, the participants with T2D had a lower DHD score and were less physically active. Fasting plasma MGO levels were higher in participants with T2D, compared with participants with NGM and participants with prediabetes. The same pattern was observed for plasma MGO levels at 120 min of the OGTT. Use of glucose-lowering, antihypertensive, and lipid-modifying drugs, was higher in individuals with T2D. Individuals with T2D had higher cell counts of neutrophils, monocytes, and each monocyte subset than the individuals without diabetes. Immune cell activation scores were not altered in participants with different glucose metabolism status.

**Table 1:** Characteristics of the study population stratified according to glucose metabolism status

Characteristic	Overall *n* = 696	NGM *n* = 376	Prediabetes *n* = 91	T2D *n* = 229	*P*-value
Sex, women (%) ^a^	361 (51.9%)	226 (60.1%)	51 (56.0%)	84 (36.7%)	< 0.001
Age (years) ^b^	60.3 ± 8.4	57.8 ± 8.3	61.5 ± 7.5	64.0 ± 7.6	< 0.001
BMI (kg/m^2^) ^b^	27.3 ± 4.7	25.7 ± 3.7	27.8 ± 4.2	29.8 ± 5.1	< 0.001
**Education status (n**,** %)**^**a**^					
Low	258 (37.1%)	94 (25.0%)	40 (44.0%)	124 (54.1)	< 0.001
Medium	186 (26.7%)	114 (30.3%)	20 (22.0%)	52 (22.7%)	< 0.001
High	252 (36.2%)	168 (44.7%)	31 (34.1%)	53 (23.1%)	< 0.001
**Smoking status (n**,** %)**^**a**^					
Never	269 (38.6%)	169 (44.9%)	31 (34.1%)	69 (30.1%)	0.004
Former	333 (47.8%)	163 (43.4%)	49 (53.8%)	121 (52.8%)	0.004
Current	94 (13.5%)	44 (11.7%)	11 (12.1%)	39 (17.0%)	0.004
**Medication use** ^**a**^					
Glucose-lowering drugs, yes (n, %)	175 (25.1%)	0 (0.0%)	0 (0.0%)	175 (76.4%)	< 0.001
Antihypertensive drugs, yes (n, %)	287 (41.2%)	79 (21.0%)	40 (44.0%)	168 (73.4%)	< 0.001
Lipid-lowering drugs, yes (n, %)	264 (37.9%)	59 (15.7%)	35 (38.5%)	170 (74.2%)	< 0.001
Systolic blood pressure (mmHg) ^b^	133.2 ± 17.8	129.3 ± 17.3	134.8 ± 16.6	139.0 ± 17.6	< 0.001
Waist circumference (cm) ^b^	96.0 ± 13.9	89.9 ± 11.3	97.8 ± 11.2	105.3 ± 13.5	< 0.001
HbA1c (mmol/mol) ^b^	40.2 ± 9.5	34.7 ± 3.9	37.7 ± 4.4	50.3 ± 9.3	< 0.001
Dutch Healthy Diet Score ^b, c^	83.6 ± 14.9	85.6 ± 14.6	84.0 ± 14.4	80.3 ± 15.1	< 0.001
Total physical activity (hours/week) ^b, c^	14.7 ± 8.4	15.6 ± 8.5	14.9 ± 7.5	12.9 ± 8.2	0.001
**Oral glucose tolerance test** ^**b**^					
T = 0 plasma MGO (nmol/L)	352.2 ± 105.7	329.9 ± 88.8	359.5 ± 100.3	385.8 ± 123.1	< 0.001
T = 120 Plasma MGO (nmol/L) ^c^	296.3 ± 95.8	257.9 ± 59.4	305.3 ± 100.8	369.0 ± 108.9	< 0.001
**Immune cell counts** ^**b**^					
Neutrophils (10^9^/L blood)	3.32 ± 1.20	2.99 ± 1.07	3.40 ± 1.21	3.82 ± 1.22	< 0.001
Monocytes (10^9^/L blood)	0.31 ± 0.11	0.29 ± 0.10	0.32 ± 0.11	0.34 ± 0.12	< 0.001
Classical monocytes (10^9^/L blood)	0.24 ± 0.09	0.23 ± 0.08	0.25 ± 0.10	0.27 ± 0.10	< 0.001
Intermediate monocytes (10^9^/L blood)	0.02 ± 0.01	0.02 ± 0.01	0.02 ± 0.01	0.03 ± 0.02	< 0.001
Non-classical monocytes (10^9^/L blood)	0.04 ± 0.02	0.04 ± 0.02	0.04 ± 0.02	0.05 ± 0.03	0.006
**Immune cell activation scores** ^**b, c**^					
Neutrophils	0.00 ± 1.00	0.03 ± 1.04	0.03 ± 0.92	−0.06 ± 0.96	0.581
Classical monocytes	0.00 ± 1.00	0.05 ± 1.12	−0.04 ± 0.76	−0.06 ± 0.86	0.434
Intermediate monocytes	0.00 ± 1.00	0.01 ± 1.21	−0.03 ± 0.65	0.00 ± 0.70	0.964
Non-classical monocytes	0.00 ± 1.00	−0.04 ± 1.09	0.02 ± 0.85	0.06 ± 0.90	0.540

Data are presented as mean ± standard deviation (SD) or group size and percentages (%). The data were analyzed with one-way ANOVA or chi-squared tests. ^a^chi-squared test. ^b^one-way ANOVA. ^c^ T = 120 plasma MGO available in *n* = 639; immune cell activation scores available in *n* = 628; Dutch healthy diet score available in *n* = 668; total physical activity available in *n* = 618. Abbreviations: NGM: normal glucose metabolism, T2D: type 2 diabetes, BMI: body mass index, HbA1c = hemoglobin A1c, MGO: methylglyoxal

### Fasting and post-OGTT plasma MGO levels and immune cell counts

Higher fasting (T = 0 min) plasma levels of MGO were significantly associated with higher numbers of neutrophils, total monocytes, and intermediate and non-classical monocytes in the age- and sex-adjusted model (model 2, Table 2). These associations were attenuated after additional adjustment for lifestyle factors, SBP, medication use, and glucose metabolism status (model 3–5, Table 2), and lost statistical significance for neutrophils and total monocytes but remained statistically significant for intermediate and non-classical monocytes. Similar positive associations were also observed for post-OGTT plasma MGO levels with numbers of neutrophils, total monocytes, and classical and intermediate monocytes in under-adjusted models (model 3 for total monocytes and classical monocytes, model 4 for neutrophils and intermediate monocytes). These associations lost statistical significance after further adjustment for SBP and medication use in model 4 for total and classical monocytes, and after further adjustment for glucose metabolism status in model 5 for neutrophils and intermediate monocytes (Table 2).

**Table 2:** Associations between plasma MGO concentrations and immune cell counts

	Model	Neutrophils	Monocytes	Classical monocytes	Intermediate monocytes	Non-classical monocytes
MGO T = 0 min (*n* = 696)	1	**0.09** **[0.02; 0.17]**	**0.11** **[0.03; 0.18]**	0.06 [−0.02; 0.13]	**0.17** **[0.09; 0.24]**	**0.11** **[0.04; 0.18]**
	2	**0.08** **[0.00; 0.15]**	**0.07** **[0.001; 0.14]**	0.03 [−0.05; 0.10]	**0.13** **[0.06; 0.21]**	**0.08** **[0.01; 0.15]**
	3	0.05 [−0.02; 0.12]	0.06 [−0.02; 0.13]	0.10 [−0.06; 0.08]	**0.12** **[0.05; 0.19]**	**0.08** **[0.07; 0.15]**
	4	0.02 [−0.05; 0.09]	0.04 [−0.03; 0.12]	−0.003 [−0.08; 0.07]	**0.10** **[0.03; 0.17]**	**0.08** **[0.01; 0.15]**
	5	0.004 [−0.07; 0.08]	0.03 [−0.04; 0.11]	−0.01 [−0.09; 0.06]	**0.09** **[0.02; 0.17]**	**0.08** **[0.002; 0.15]**
MGO T = 120 min (*n* = 639)	1	**0.21** **[0.13; 0.28]**	**0.18** **[0.10; 0.26]**	**0.15** **[0.08; 0.23]**	**0.21** **[0.13; 0.29]**	**0.13** **[0.05; 0.21]**
	2	**0.18** **[0.11; 0.26]**	**0.12** **[0.04; 0.19]**	**0.10** **[0.02; 0.18]**	**0.15** **[0.08; 0.23]**	0.07 [−0.01; 0.15]
	3	**0.15** **[0.07; 0.23]**	**0.09** **[0.02; 0.17]**	**0.08** **[0.001; 0.16]**	**0.13** **[0.05; 0.20]**	0.07 [−0.01; 0.14]
	4	**0.12** **[0.03; 0.20]**	0.08 [−0.01; 0.16]	0.07 [−0.02; 0.15]	**0.10** **[0.07; 0.18]**	0.07 [−0.01; 0.16]
	5	0.07 [−0.02; 0.16]	0.05 [−0.04; 0.14]	0.04 [−0.05; 0.13]	0.09 [−0.001; 0.18]	0.06 [−0.03; 0.15]

Data are presented as regression coefficients (β) and 95% CI. Plasma MGO concentrations and numbers of immune cells (neutrophils, monocytes, and monocyte subsets: classical, intermediate, and non-classical monocytes) were ln-transformed to ensure normality and then standardized. β [95% CI] represents 1-SD difference in immune cell counts per 1-SD increase of plasma MGO levels. Model 1: crude model; Model 2: adjusted for age and sex; Model 3: model 2 + adjusted for BMI, education status, and smoking status; Model 4: model 3 + adjusted for SBP, glucose lowering drugs, antihypertensive drugs, lipid lowering drugs; Model 5: model 4 + adjusted for glucose metabolism status. Abbreviations: MGO, methylglyoxal; SBP, systolic blood pressure. BMI, body mass index

### Fasting and post-OGTT plasma MGO levels and immune cell activation

A significant association was only found for higher fasting plasma levels of MGO with lower activation scores of intermediate monocytes in the fully adjusted model (Table 3), but not of neutrophils, classical, and non-classical monocytes. With regard to the individual activation markers, higher fasting plasma MGO levels were significantly associated with lower expression of CD11c on all monocyte subsets, and with lower CX3CR1 surface expression on intermediate monocytes in fully adjusted analyses (Supplementary Tables 1–4). For post-OGTT plasma levels of MGO, no association was found with the activation scores of any of the immune cell subsets (Table 3). Analyses with individual activation markers also showed no association with post-OGTT plasma MGO levels (Supplementary Tables 1–4).

**Table 3:** Associations between plasma MGO concentrations and immune cell activation scores

	Model	Neutrophils	Classical monocytes	Intermediate monocytes	Non-classical monocytes
MGO T = 0 min (*n* = 628)	1	−0.03 [−0.11; 0.05]	**−0.09** **[−0.16; −0.01]**	**−0.13** **[−0.21; −0.06]**	−0.03 [−0.11; 0.04]
	2	−0.02 [−0.10; 0.06]	−0.08 [−0.16; 0.002]	**−0.13** **[−0.21; −0.05]**	−0.02 [−0.10; 0.06]
	3	−0.03 [−0.11; 0.05]	−0.07 [−0.15; 0.01]	**−0.13** **[−0.21; −0.05]**	−0.03 [−0.11; 0.05]
	4	−0.01 [−0.09; 0.07]	−0.08 [−0.16; 0.01]	**−0.14** **[−0.22; −0.06]**	−0.04 [−0.12; 0.21]
	5	−0.01 [−0.10; 0.07]	−0.07 [−0.15; 0.01]	**−0.14** **[−0.22; −0.06]**	−0.05 [−0.13; 0.03]
MGO T = 120 min (*n* = 576)	1	−0.04 [−0.13; 0.04]	−0.01 [−0.09; 0.07]	−0.01 [−0.09; 0.07]	−0.01 [−0.09; 0.07]
	2	−0.04 [−0.12; 0.01]	0.002 [−0.08; 0.09]	−0.01 [−0.09; 0.08]	0.03 [−0.05; 0.11]
	3	−0.04 [−0.13; 0.04]	0.01 [−0.08; 0.10]	0.00 [−0.09; 0.09]	0.02 [−0.07; 0.10]
	4	−0.03 [−0.12; 0.06]	−0.01 [−0.11; 0.08]	−0.02 [−0.12; 0.07]	−0.01 [−0.10; 0.08]
	5	−0.05 [−0.15; 0.05]	0.003 [−0.10; 0.10]	−0.03 [−0.13; 0.07]	−0.02 [−0.12; 0.08]

Data are presented as β [95% CI]. Plasma MGO concentrations were ln-transformed to ensure normality and then standardized. β [95% CI] represents 1-SD difference in immune cell activation scores per 1-SD increase of plasma MGO levels. Model 1: crude model; Model 2: adjusted for age and sex; Model 3: model 2 + adjusted for BMI, education status, and smoking status; Model 4: model 3 + adjusted for SBP, glucose lowering drugs, antihypertensive drugs, lipid lowering drugs; Model 5: model 4 + adjusted for glucose metabolism status. Abbreviations: MGO, methylglyoxal; SBP, systolic blood pressure. BMI, body mass index

### Interaction analyses

No significant interactions were observed with sex for the analyses between fasting and post-OGTT plasma MGO levels with immune cell counts or immune cell activation (*p*_interaction_ > 0.05). Significant interactions with T2D were shown for the analyses of fasting plasma MGO levels with counts of total and classical monocytes (*p*_interaction_ = 0.046 and 0.039, respectively). Stratified analyses revealed that higher fasting plasma levels of MGO were significantly associated with more total and classical monocytes in participants with T2D after adjustment for age and sex, but with less classical monocytes in participants with NGM after adjustment for age, sex, and lifestyle. However, these associations became nonsignificant after full adjustment (Table 4).

**Table 4:** Associations between plasma MGO concentrations and immune cell counts, stratified for glucose metabolism status

	Model	Neutrophils	Monocytes	Classical monocytes	Intermediate monocytes	Non-classical monocytes
MGO T = 0 min						
NGM (*n* = 376)	1	−0.06 [−0.17; 0.04]	−0.05 [−0.15; 0.06]	−0.10 [−0.20; 0.004]	0.06 [−0.04; 0.16]	0.03 [−0.07; 0.13]
	2	−0.07 [−0.17; 0.04]	−0.05 [−0.15; 0.05]	−0.10 [−0.20; −0.004]	0.05 [−0.05; 0.15]	0.02 [−0.08; 0.12]
	3	−0.06 [−0.16; 0.04]	−0.05 [−0.15; 0.05]	−0.10 [−0.20; −0.002]	0.05 [−0.05; 0.15]	0.02 [−0.08; 0.11]
	4	−0.06 [−0.16; 0.05]	−0.05 [−0.15; 0.05]	−0.10 [−0.20; 0.003]	0.05 [−0.05; 0.15]	0.02 [−0.08; 0.12]
Prediabetes (*n* = 91)	1	0.03 [−0.18; 0.24]	0.17 [−0.04; 0.38]	0.15 [−0.06; 0.35]	0.13 [−0.08; 0.34]	0.18 [−0.03; 0.38]
	2	0.03 [−0.19; 0.24]	0.15 [−0.06; 0.35]	0.12 [−0.09; 0.33]	0.11 [−0.10; 0.32]	0.16 [−0.05; 0.36]
	3	0.04 [−0.18; 0.26]	0.16 [−0.06; 0.37]	0.14 [−0.07; 0.36]	0.09 [−0.13; 0.31]	0.13 [−0.09; 0.34]
	4	−0.02 [−0.25; 0.21]	0.12 [−0.10; 0.34]	0.11 [−0.11; 0.33]	0.01 [−0.21; 0.23]	0.12 [−0.09; 0.34]
T2D (*n* = 229)	1	**0.15** **[0.02; 0.28]**	**0.14** **[0.01; 0.27]**	0.09 [−0.04; 0.22]	**0.17** **[0.04; 0.30]**	0.12 [−0.01; 0.25]
	2	**0.15** **[0.02; 0.28]**	**0.14** **[0.01; 0.26]**	0.09 [−0.04; 0.22]	**0.17** **[0.04; 0.30]**	0.12 [−0.01; 0.25]
	3	0.12 [−0.01; 0.24]	0.11 [−0.01; 0.24]	0.06 [−0.06; 0.19]	**0.16** **[0.03; 0.29]**	**0.13** **[0.01; 0.26]**
	4	0.12 [−0.004; 0.24]	0.12 [−0.01; 0.25]	0.07 [−0.06; 0.20]	**0.17** **[0.04; 0.30]**	**0.14** **[0.01; 0.27]**
**MGO T = 120 min**						
NGM (*n* = 367)	1	0.08 [−0.02; 0.18]	0.05 [−0.05; 0.15]	0.05 [−0.05; 0.16]	0.06 [−0.05; 0.16]	0.04 [−0.06; 0.14]
	2	0.08 [−0.02; 0.18]	0.04 [−0.06; 0.14]	0.04 [−0.06; 0.14]	0.04 [−0.06; 0.14]	0.02 [−0.08; 0.12]
	3	0.08 [−0.02; 0.18]	0.04 [−0.06; 0.14]	0.04 [−0.06; 0.14]	0.06 [−0.04; 0.16]	0.03 [−0.07; 0.13]
	4	0.09 [−0.02; 0.19]	0.04 [−0.06; 0.14]	0.04 [−0.06; 0.14]	0.06 [−0.04; 0.16]	0.04 [−0.06; 0.14]
Prediabetes (*n* = 89)	1	0.06 [−0.15; 0.28]	0.02 [−0.19; 0.24]	0.01 [−0.20; 0.22]	0.03 [−0.18; 0.24]	0.07 [−0.14; 0.29]
	2	0.07 [−0.15; 0.28]	0.04 [−0.17; 0.25]	0.02 [−0.19; 0.24]	0.04 [−0.17; 0.25]	0.09 [−0.12; 0.30]
	3	0.12 [−0.11; 0.34]	0.10 [−0.12; 0.31]	0.08 [−0.13; 0.30]	0.08 [−0.14; 0.30]	0.10 [−0.12; 0.31]
	4	0.08 [−0.15; 0.31]	0.04 [−0.18; 0.27]	0.03 [−0.20; 0.26]	0.02 [−0.20; 0.24]	0.10 [−0.12; 0.32]
T2D (*n* = 183)	1	−0.02 [−0.17; 0.13]	0.08 [−0.06; 0.23]	0.04 [−0.11; 0.19]	**0.15** **[0.01; 0.30]**	0.09 [−0.06; 0.23]
	2	−0.03 [−0.17; 0.12]	0.05 [−0.09; 0.19]	0.01 [−0.13; 0.15]	0.13 [−0.01; 0.28]	0.05 [−0.09; 0.19]
	3	−0.04 [−0.18; 0.11]	0.07 [−0.08; 0.21]	0.02 [−0.12; 0.16]	**0.15** **[0.01; 0.30]**	0.07 [−0.07; 0.21]
	4	−0.04 [−0.18; 0.11]	0.07 [−0.08; 0.21]	0.02 [−0.12; 0.16]	0.15 [−0.001; 0.29]	0.07 [−0.07; 0.21]

Data are presented as regression coefficients (β) and 95% CI. Plasma MGO concentrations and numbers of immune cells (neutrophils, monocytes, and monocyte subsets: classical, intermediate, and non-classical monocytes) were ln-transformed to ensure normality and then standardized. β [95% CI] represents 1-SD difference in immune cell counts per 1-SD increase of plasma MGO levels. Model 1: crude model; Model 2: adjusted for age and sex; Model 3: model 2 + adjusted for BMI, education status, and smoking status; Model 4: model 3 + adjusted for SBP, glucose lowering drugs, antihypertensive drugs, lipid lowering drugs. Abbreviations: MGO, methylglyoxal; SBP, systolic blood pressure. BMI, body mass index. NGM, normal glucose metabolism; T2D, type 2 diabetes

Glucose metabolism status was considered to be not just a potential confounder, but also a potential mediator and collider in the analyses, which may result in overadjustment in the model. Therefore, stratified analyses were also performed for associations between fasting plasma MGO and counts of neutrophils, intermediate and non-classical monocytes, as well as associations between post-OGTT plasma MGO levels and counts of all immune cell subsets. Statistically significant associations were found for higher fasting plasma MGO levels and higher numbers of intermediate and non-classical monocytes in participants with T2D after full adjustment. In the same population, positive associations were also observed for fasting plasma MGO levels with numbers of neutrophils after adjustment for age and sex, and post-OGTT plasma MGO levels with numbers of intermediate monocytes after adjustment for age, sex, and lifestyle. These associations lost statistical significance in fully adjusted analyses (Table 4).

### Sensitivity analyses

Associations of fasting and post-OGTT plasma MGO levels with immune cell counts or activation were not materially changed in the sensitivity analyses by replacing BMI with waist circumference in the model (Supplementary Tables 5, 6). Associations also remained similar when we added DHD and physical activity to the model. However, excluding the missing data of DHD (*n* = 51–55) and physical activity (*n* = 113–126) resulted in a loss of statistical significance for the associations of higher fasting plasma MGO levels with higher numbers of intermediate and non-classical monocytes, and with lower activation scores of intermediate monocytes (Supplementary Tables 5, 6).

## Discussion

In this cross-sectional study, we investigated the associations of fasting and post-OGTT plasma MGO concentrations with the numbers and activation of circulating immune cell subsets in a population-based cohort. Our data showed that higher fasting plasma levels of MGO were associated with more intermediate and non-classical monocytes, but with less activation of intermediate monocytes. No consistent associations were shown for post-OGTT plasma MGO levels with either immune cell counts or activation.

We found higher plasma MGO levels both before and after an OGTT in T2D compared to individuals with prediabetes and NGM. This is in agreement with several other studies [1, 2, 8]. Simultaneously, we found that individuals with T2D also have higher cell counts of total monocytes and neutrophils at fasting state. Another study, however, did not find a significant difference of total monocyte counts between T2D subjects and controls [33], which is most likely due to lack of power of that study.

In the current study, the crude associations between plasma MGO levels and both neutrophil and total monocyte counts were positive and significant. In a previous publication, higher MGO concentrations of COVID-19 patients who died in intensive care unit were correlated with higher total monocyte numbers [34]. However, these analyses were performed without any adjustment. In our current study, statistical significance of the associations was lost after adjustment for several potential confounders. Therefore, the relation between plasma MGO levels and monocytes necessitates further validation in other population-based cohort studies. In our stratified analyses, a possible relationship was shown for higher fasting plasma MGO levels and more neutrophils (*p* = 0.057) and total monocytes (*p* = 0.06) in participants with T2D. Several experimental studies have previously shown that MGO can enhance leukocyte recruitment. Exogenous administration of MGO in mice led to enhanced leukocyte recruitment via endothelial activation [35–37]. Incubating MGO with human endothelial cells ex vivo led to an increase of monocyte adhesion and transmigration [23]. The correlation between MGO elevation and endothelial activation has been demonstrated in a 12-year follow up study [38]. Earlier data from The Maastricht Study also support a positive association between plasma MGO levels and endothelial activation, particularly with the biomarker soluble E-selectin [2]. E-selectin is expressed exclusively on skin microvascular endothelial cells under baseline conditions, but can also be rapidly induced on other endothelial cells in response to inflammatory cytokines [39]. These findings together suggest that endothelial activation may play an important role in regulating the MGO-induced leukocytes recruitment in the circulation, and that E-selectin-mediated signaling pathway might be involved in this process. Moreover, a recent mouse study further suggests that MGO spikes may increase myeloid cell production by promoting myelopoiesis [9]. In addition to the effect on leukocyte-endothelial interactions, MGO also has direct effects on immune cell function. Ex vivo exposure to MGO led to neutrophil activation and increased pro-inflammatory cytokine release, and simultaneously also apoptosis and phagocytosis dysfunction of neutrophils [21, 40, 41]. In monocytes, MGO treatment triggered apoptosis and oxidative stress [42], and in combination with lipopolysaccharide (LPS) induced nuclear translocation of hypoxia inducible factor (HIF)−1α in the U937 cell line [43]. However, our data do not support a relationship between plasma MGO levels and the activation of circulating total neutrophils and/or monocytes.

Since human monocytes are heterogeneous and have different functions of each subsets [44], we also explored the associations of plasma MGO levels with monocyte subsets. We found a significant association between higher fasting plasma MGO levels and higher numbers of intermediate and non-classical monocytes, and an association of higher post-OGTT plasma levels of MGO with higher intermediate monocytes (*p* = 0.051). Stratified analyses showed that these associations appear only in participants with T2D. Plenty of studies have revealed a link between circulating monocyte subsets, especially intermediate and non-classical monocytes, and clinical outcome of cardiovascular diseases [14]. Positive correlations have been shown for intermediate monocytes with intima-media thickness [45, 46], coronary plaque rupture [47, 48], unstable angina [49], adhesion mediated by vascular cell adhesion molecule-1 [50], coronary artery calcification [51], restenosis after peripheral percutaneous transluminal angioplasty [52], as well as cardiovascular events prediction [53–55]. Similarly, non-classical monocytes are positively related to intima-media thickness [46] and coronary plaque rupture [47, 48]. In addition to these elevated numbers of monocyte subsets, increased plasma MGO levels have been indicated to contribute to the development of cardiovascular diseases [2, 3, 9]. Our findings support an interaction between circulating MGO and intermediate and non-classical monocytes in individuals with T2D, which may partly explain the increased risk of cardiovascular disease in people with T2D. Interestingly, our data revealed an association between fasting plasma MGO levels and lower activation of circulating intermediate monocytes, mainly due to lower expression of CD11c and CX3CR1. A previous microarray study observed a severe down-regulation of CX3CR1 expression on circulating monocytes from septic shock patients and was suggested to be a feature of sepsis-induced immunosuppression [56]. It has been confirmed that repetitive inflammatory stimulation can result in exhausted monocytes [57]. A similar phenomenon was also shown in individuals with T2D, who have an impaired inflammatory response of monocytes, as reflected by suppressed TNF secretion and CD11b expression in response to LPS challenge [58]. Thus, our findings suggest a relationship between higher plasma MGO levels and intermediate monocyte exhaustion. Our result also may point to a relation between fasting plasma MGO levels and classical and non-classical monocyte exhaustion, although this was only based on a lower CD11c expression, but not total activation scores. Further studies are needed to validate these findings.

The present study has several strengths. We are the first to investigate the association of fasting and post-OGTT plasma MGO levels with the circulating immune cells counts and activation in a population-based cohort. Our study provides the first epidemiological evidence for the interaction between plasma MGO and circulating immune cells in people with and without T2D. The comprehensive phenotyping and population-based approach of The Maastricht Study enabled us to adjust for multiple potential confounders in the linear regression analyses. In addition, gold standard methods were applied for the quantifications of plasma MGO levels and immune cells, where the highly specific UPLC-MS/MS technique and flow cytometry were used, respectively. Flow cytometry performed on fresh blood enabled us to measure also neutrophils, as well as several activation markers on the surface of the immune cells. The combination of flow cytometry and automated cell counter allowed us to assess the actual counts of not only the total population of neutrophils and monocytes, but also the subsets of monocytes in the peripheral blood.

The present study also has several limitations. First, immune cells from peripheral blood were only measured at baseline at a relatively resting state, which may lead to an underestimation of the associations with immune cell activation. The use of fasting blood may mask the “real” monocyte number since fasting in humans is linked to monocyte retention in the bone marrow [59]. Postprandial immune cells should also be included in future research as they are maybe more active after glucose spikes [10]. Second, although post-OGTT plasma MGO levels were shown to associate with T2D and its vascular complications [2, 8], the MGO concentrations at 120 min of the OGTT may not be the best time point to estimate the postprandial changes of MGO and based on our previous findings [8], earlier time points may better reflect the MGO peak levels. This may partly explain the inconsistent findings for the post-OGTT plasma MGO levels in the current study. Furthermore, the assessment of immune cell activation includes, but is not limited to, the markers used in the current study. Therefore, we cannot fully exclude the possibility that associations may be observed between plasma MGO levels and other immune cell activation markers, such as neutrophil elastase and myeloperoxidase [60]. Lastly, due to the cross-sectional nature of the study, assessment of a causal relationship was not possible. Although it has been suggested that excess MGO can cause an increase in monocyte numbers [20], the possibility that MGO accumulation in the plasma is a consequence of abnormal changes in immune cells cannot be ruled out. The mystery of the causal link between MGO levels and immune cell changes remains to be explored in future research.

In conclusion, higher fasting but not post-OGTT plasma MGO concentrations are associated with higher numbers of circulating intermediate and non-classical monocytes, and with lower activation of intermediate monocytes. These findings support a potential interaction between plasma MGO and circulating intermediate monocytes, as a possible contributor to the increased risk of cardiovascular disease in individuals with type 2 diabetes.

## Electronic supplementary material

Below is the link to the electronic supplementary material.

Supplementary Material 1

Supplementary Material 2

## References

[1] Schalkwijk CG, Stehouwer CDA (2020) Methylglyoxal, a Highly Reactive Dicarbonyl Compound, in Diabetes, Its Vascular Complications, and Other Age-Related Diseases. Physiol Rev. 100(1):407– 61. 10.1152/physrev.00001.2019 10.1152/physrev.00001.2019 31539311

[2] Hanssen N, Scheijen J, Houben A et al (2021) Fasting and post-oral-glucose-load levels of methylglyoxal are associated with microvascular, but not macrovascular, disease in individuals with and without (pre) diabetes: the Maastricht Study. Diabetes Metab 47(1):101148. 10.1016/j.diabet.2020.02.002 32058030 10.1016/j.diabet.2020.02.002

[3] Hanssen NM, Westerink J, Scheijen JL et al (2018) Higher plasma methylglyoxal levels are associated with incident cardiovascular disease and mortality in individuals with type 2 diabetes. Diabetes Care 41(8):1689–1695. 10.2337/dc18-0159 29784769 10.2337/dc18-0159

[4] Hanssen NM, Kraakman MJ, Flynn MC, Nagareddy PR, Schalkwijk CG, Murphy AJ (2020) Postprandial glucose spikes, an important contributor to cardiovascular disease in diabetes? Frontiers in cardiovascular medicine. 7:168. 10.3389/fcvm.2020.570553 10.3389/fcvm.2020.570553 PMC7530333 33195459

[5] Price CL, Hassi HO, English NR, Blakemore AI, Stagg AJ, Knight SC (2010) Methylglyoxal modulates immune responses: relevance to diabetes. J Cell Mol Med 14(6B):1806–1815. 10.1111/j.1582-4934.2009.00803.x 19538479 10.1111/j.1582-4934.2009.00803.x PMC3829040

[6] Hanssen NM, Wouters K, Huijberts MS et al (2014) Higher levels of advanced glycation endproducts in human carotid atherosclerotic plaques are associated with a rupture-prone phenotype. Eur Heart J 35(17):1137–1146. 10.1093/eurheartj/eht402 24126878 10.1093/eurheartj/eht402

[7] Maessen DE, Hanssen NM, Lips MA et al (2016) Energy restriction and Roux-en-Y gastric bypass reduce postprandial α-dicarbonyl stress in obese women with type 2 diabetes. Diabetologia 59(9):2013–2017 27312699 10.1007/s00125-016-4009-1 PMC4969347

[8] Maessen DE, Hanssen NM, Scheijen JL et al (2015) Post–glucose load plasma α-dicarbonyl concentrations are increased in individuals with impaired glucose metabolism and type 2 diabetes: the CODAM study. Diabetes Care 38(5):913–920 25710921 10.2337/dc14-2605

[9] Hanssen NM, Tikellis C, Pickering RJ et al (2023) Pyridoxamine prevents increased atherosclerosis by intermittent methylglyoxal spikes in the aortic arches of ApoE-/-mice. Biomed Pharmacother 158:114211 36916437 10.1016/j.biopha.2022.114211

[10] Flynn MC, Kraakman MJ, Tikellis C et al (2020) Transient intermittent hyperglycemia accelerates atherosclerosis by promoting myelopoiesis. Circul Res 127(7):877–892. 10.1161/CIRCRESAHA.120.316653 10.1161/CIRCRESAHA.120.316653 PMC7486277 32564710

[11] Kanter JE, Hsu CC, Bornfeldt KE (2020) Monocytes and macrophages as protagonists in Vascular complications of Diabetes. Front Cardiovasc Med 7:10. 10.3389/fcvm.2020.00010 32118048 10.3389/fcvm.2020.00010 PMC7033616

[12] Keeter WC, Moriarty AK, Galkina EV (2021) Role of neutrophils in type 2 diabetes and associated atherosclerosis. Int J Biochem Cell Biol 141:106098. 10.1016/j.biocel.2021.106098 34655814 10.1016/j.biocel.2021.106098 PMC8962624

[13] Berbudi A, Rahmadika N, Tjahjadi AI, Ruslami R (2020) Type 2 diabetes and its impact on the Immune System. Curr Diabetes Rev 16(5):442–449. 10.2174/1573399815666191024085838 31657690 10.2174/1573399815666191024085838 PMC7475801

[14] Biessen EA, Wouters K (2017) Macrophage complexity in human atherosclerosis: opportunities for treatment? Current opinion in lipidology. 28(5):419–426. 10.1097/MOL.0000000000000447 10.1097/MOL.0000000000000447 28759472

[15] Li Y, Ma Q, Li P et al (2020) Proteomics reveals different pathological processes of adipose tissue, liver, and skeletal muscle under insulin resistance. J Cell Physiol 235(10):6441–6461. 10.1002/jcp.29658 32115712 10.1002/jcp.29658

[16] Ruder AV, Wetzels SM, Temmerman L, Biessen EA, Goossens P (2023) Monocyte heterogeneity in cardiovascular disease. Cardiovascular Res 119(11):2033–2045 10.1093/cvr/cvad069 PMC10478755 37161473

[17] Wouters K, Gaens K, Bijnen M et al (2017) Circulating classical monocytes are associated with CD11c(+) macrophages in human visceral adipose tissue. Sci Rep 7(1):42665. 10.1038/srep42665 28198418 10.1038/srep42665 PMC5309742

[18] Ziegler-Heitbrock L, Ancuta P, Crowe S et al (2010) Nomenclature of monocytes and dendritic cells in blood. Blood J Am Soc Hematol 116(16):e74–e80 10.1182/blood-2010-02-258558 20628149

[19] Sprangers S, Vries TJd, Everts V (2016) Monocyte heterogeneity: consequences for monocyte-derived immune cells. Journal of immunology research. 2016(1):1475435 10.1155/2016/1475435 PMC4958468 27478854

[20] Zhang X, Schalkwijk CG, Wouters K (2022) Immunometabolism and the modulation of immune responses and host defense: a role for methylglyoxal? Biochimica et Biophysica Acta (BBA)-Molecular basis of Disease. 1868(8):166425. 10.1016/j.bbadis.2022.166425 10.1016/j.bbadis.2022.166425 35500827

[21] Wang H, Meng QH, Gordon JR, Khandwala H, Wu L (2007) Proinflammatory and proapoptotic effects of methylglyoxal on neutrophils from patients with type 2 diabetes mellitus. Clin Biochem 40(16–17):1232–1239. 10.1016/j.clinbiochem.2007.07.016 17825811 10.1016/j.clinbiochem.2007.07.016

[22] Dorenkamp M, Muller JP, Shanmuganathan KS et al (2018) Hyperglycaemia-induced methylglyoxal accumulation potentiates VEGF resistance of diabetic monocytes through the aberrant activation of tyrosine phosphatase SHP-2/SRC kinase signalling axis. Sci Rep 8(1):14684. 10.1038/s41598-018-33014-9 30279491 10.1038/s41598-018-33014-9 PMC6168515

[23] Rom S, Heldt NA, Gajghate S, Seliga A, Reichenbach NL, Persidsky Y (2020) Hyperglycemia and advanced glycation end products disrupt BBB and promote occludin and claudin-5 protein secretion on extracellular microvesicles. Sci Rep 10(1):7274. 10.1038/s41598-020-64349-x 32350344 10.1038/s41598-020-64349-x PMC7190636

[24] Schram MT, Sep SJ, van der Kallen CJ et al (2014) The Maastricht Study: an extensive phenotyping study on determinants of type 2 diabetes, its complications and its comorbidities. Eur J Epidemiol 29(6):439–451. 10.1007/s10654-014-9889-0 24756374 10.1007/s10654-014-9889-0

[25] Organization WH (2006) Definition and diagnosis of diabetes mellitus and intermediate hyperglycaemia. report of a WHO/IDF consultation

[26] Scheijen JL, Schalkwijk CG (2014) Quantification of glyoxal, methylglyoxal and 3-deoxyglucosone in blood and plasma by ultra performance liquid chromatography tandem mass spectrometry: evaluation of blood specimen. Clin Chem Lab Med 52(1):85–91 23492564 10.1515/cclm-2012-0878

[27] Connelly AN, Huijbregts RP, Pal HC et al (2022) Optimization of methods for the accurate characterization of whole blood neutrophils. Sci Rep 12(1):3667 35256648 10.1038/s41598-022-07455-2 PMC8901620

[28] Le Joncour A, Régnier P, Maciejewski-Duval A et al (2023) Reduction of Neutrophil activation by phosphodiesterase 4 blockade in Behçet’s Disease. Arthritis Rheumatol 75(9):1628–1637 36862398 10.1002/art.42486

[29] Al-Rashed F, Ahmad Z, Snider AJ et al (2021) Ceramide kinase regulates TNF-α-induced immune responses in human monocytic cells. Sci Rep 11(1):8259 33859296 10.1038/s41598-021-87795-7 PMC8050074

[30] Janssen E, Kohler S, Geraets AFJ et al (2021) Low-grade inflammation and endothelial dysfunction predict four-year risk and course of depressive symptoms: the Maastricht study. Brain Behav Immun 97:61–67. 10.1016/j.bbi.2021.06.013 34186200 10.1016/j.bbi.2021.06.013

[31] Hekler EB, Buman MP, Haskell WL et al (2012) Reliability and validity of CHAMPS self-reported sedentary-to-vigorous intensity physical activity in older adults. J Phys Activity Health 9(2):225–236 10.1123/jpah.9.2.225 PMC4733646 22368222

[32] Looman M, Feskens EJ, de Rijk M et al (2017) Development and evaluation of the Dutch healthy Diet index 2015. Public Health Nutr 20(13):2289–2299 28625202 10.1017/S136898001700091X PMC10261559

[33] Menart-Houtermans B, Rutter R, Nowotny B et al (2014) Leukocyte profiles differ between type 1 and type 2 diabetes and are associated with metabolic phenotypes: results from the German diabetes study (GDS). Diabetes Care 37(8):2326–2333. 10.2337/dc14-0316 25061140 10.2337/dc14-0316

[34] Alomar FA, Alshakhs MN, Abohelaika S et al (2022) Elevated plasma level of the glycolysis byproduct methylglyoxal on admission is an independent biomarker of mortality in ICU COVID-19 patients. Sci Rep 12(1):9510. 10.1038/s41598-022-12751-y 35680931 10.1038/s41598-022-12751-y PMC9178541

[35] Su Y, Lei X, Wu L, Liu L (2012) The role of endothelial cell adhesion molecules P-selectin, E-selectin and intercellular adhesion molecule-1 in leucocyte recruitment induced by exogenous methylglyoxal. Immunology 137(1):65–79. 10.1111/j.1365-2567.2012.03608.x 22681228 10.1111/j.1365-2567.2012.03608.x PMC3449248

[36] Su Y, Qadri SM, Cayabyab FS, Wu L, Liu L (2014) Regulation of methylglyoxal-elicited leukocyte recruitment by endothelial SGK1/GSK3 signaling. Biochim Biophys Acta 1843(11):2481–2491. 10.1016/j.bbamcr.2014.06.018 25003317 10.1016/j.bbamcr.2014.06.018

[37] Su Y, Qadri SM, Hossain M, Wu L, Liu L (2013) Uncoupling of eNOS contributes to redox-sensitive leukocyte recruitment and microvascular leakage elicited by methylglyoxal. Biochem Pharmacol 86(12):1762–1774. 10.1016/j.bcp.2013.10.008 24144633 10.1016/j.bcp.2013.10.008

[38] Hanssen NM, Scheijen JL, Jorsal A et al (2017) Higher plasma methylglyoxal levels are associated with incident cardiovascular disease in individuals with type 1 diabetes: a 12-year follow-up study. Diabetes 66(8):2278–2283 28588100 10.2337/db16-1578

[39] Ley K (2003) The role of selectins in inflammation and disease. Trends Mol Med 9(6):263–268 12829015 10.1016/s1471-4914(03)00071-6

[40] Guerra BA, Bolin AP, Otton R (2012) Carbonyl stress and a combination of astaxanthin/vitamin C induce biochemical changes in human neutrophils. Toxicol Vitro 26(7):1181–1190. 10.1016/j.tiv.2012.06.010 10.1016/j.tiv.2012.06.010 22750055

[41] Ward RA, McLeish KR (2004) Methylglyoxal: a stimulus to neutrophil oxygen radical production in chronic renal failure? Nephrol Dial Transpl 19(7):1702–1707. 10.1093/ndt/gfh271 10.1093/ndt/gfh271 15150351

[42] Okado A, Kawasaki Y, Hasuike Y et al (1996) Induction of apoptotic cell death by methylglyoxal and 3-deoxyglucosone in macrophage-derived cell lines. Biochem Biophys Res Commun 225(1):219–224. 10.1006/bbrc.1996.1157 8769121 10.1006/bbrc.1996.1157

[43] Iacobini C, Vitale M, Pugliese G, Menini S (2021) Normalizing HIF-1alpha signaling improves Cellular glucose metabolism and blocks the pathological pathways of hyperglycemic damage. Biomedicines 9(9):1139. 10.3390/biomedicines9091139 34572324 10.3390/biomedicines9091139 PMC8471680

[44] Kapellos TS, Bonaguro L, Gemünd I et al (2019) Human monocyte subsets and phenotypes in major chronic inflammatory diseases. Frontiers in immunology.2035 10.3389/fimmu.2019.02035 PMC6728754 31543877

[45] Poitou C, Dalmas E, Renovato M et al (2011) CD14dimCD16 + and CD14 + CD16 + monocytes in obesity and during weight loss: relationships with fat mass and subclinical atherosclerosis. Arterioscler Thromb Vasc Biol 31(10):2322–2330. 10.1161/ATVBAHA.111.230979 21799175 10.1161/ATVBAHA.111.230979

[46] Rogacev KS, Ulrich C, Blomer L et al (2010) Monocyte heterogeneity in obesity and subclinical atherosclerosis. Eur Heart J 31(3):369–376. 10.1093/eurheartj/ehp308 19687164 10.1093/eurheartj/ehp308

[47] Imanishi T, Ikejima H, Tsujioka H et al (2010) Association of monocyte subset counts with coronary fibrous cap thickness in patients with unstable angina pectoris. Atherosclerosis 212(2):628–635. 10.1016/j.atherosclerosis.2010.06.025 20615506 10.1016/j.atherosclerosis.2010.06.025

[48] Ikejima H, Imanishi T, Tsujioka H et al (2010) Upregulation of fractalkine and its receptor, CX3CR1, is associated with coronary plaque rupture in patients with unstable angina pectoris. Circ J 74(2):337–345 20019415 10.1253/circj.cj-09-0484

[49] Zeng S, Zhou X, Ge L et al (2014) Monocyte subsets and monocyte-platelet aggregates in patients with unstable angina. J Thromb Thrombolysis 38(4):439–446. 10.1007/s11239-014-1083-4 24844803 10.1007/s11239-014-1083-4

[50] Foster GA, Gower RM, Stanhope KL, Havel PJ, Simon SI, Armstrong EJ (2013) On-chip phenotypic analysis of inflammatory monocytes in atherogenesis and myocardial infarction. Proc Natl Acad Sci U S A 110(34):13944–13949. 10.1073/pnas.1300651110 23918401 10.1073/pnas.1300651110 PMC3752270

[51] Winchester R, Giles JT, Nativ S et al (2016) Association of Elevations of Specific T Cell and monocyte subpopulations in rheumatoid arthritis with subclinical coronary artery atherosclerosis. Arthritis Rheumatol 68(1):92–102. 10.1002/art.39419 26360530 10.1002/art.39419 PMC4690807

[52] Wildgruber M, Czubba M, Aschenbrenner T et al (2016) Increased intermediate CD14(++)CD16(++) monocyte subset levels associate with restenosis after peripheral percutaneous transluminal angioplasty. Atherosclerosis 253:128–134. 10.1016/j.atherosclerosis.2016.09.002 27615596 10.1016/j.atherosclerosis.2016.09.002

[53] Heine GH, Ulrich C, Seibert E et al (2008) CD14(++)CD16 + monocytes but not total monocyte numbers predict cardiovascular events in dialysis patients. Kidney Int 73(5):622–629. 10.1038/sj.ki.5002744 18160960 10.1038/sj.ki.5002744

[54] Rogacev KS, Cremers B, Zawada AM et al (2012) CD14 + + CD16 + monocytes independently predict cardiovascular events: a cohort study of 951 patients referred for elective coronary angiography. J Am Coll Cardiol 60(16):1512–1520. 10.1016/j.jacc.2012.07.019 22999728 10.1016/j.jacc.2012.07.019

[55] Rogacev KS, Seiler S, Zawada AM et al (2011) CD14 + + CD16 + monocytes and cardiovascular outcome in patients with chronic kidney disease. Eur Heart J 32(1):84–92. 10.1093/eurheartj/ehq371 20943670 10.1093/eurheartj/ehq371

[56] Pachot A, Cazalis M-A, Venet F et al (2008) Decreased expression of the fractalkine receptor CX3CR1 on circulating monocytes as new feature of sepsis-induced immunosuppression. J Immunol 180(9):6421–6429 18424766 10.4049/jimmunol.180.9.6421

[57] Pradhan K, Yi Z, Geng S, Li L (2021) Development of exhausted memory monocytes and underlying mechanisms. Front Immunol 12:778830. 10.3389/fimmu.2021.778830 34777396 10.3389/fimmu.2021.778830 PMC8583871

[58] Khondkaryan L, Margaryan S, Poghosyan D, Manukyan G (2018) Impaired inflammatory response to LPS in type 2 diabetes Mellitus. Int J Inflam 2018(2157434). 10.1155/2018/2157434 10.1155/2018/2157434 PMC5820544 29568481

[59] Jordan S, Tung N, Casanova-Acebes M et al (2019) Dietary intake regulates the circulating inflammatory Monocyte Pool. Cell 178(5):1102–14e17. 10.1016/j.cell.2019.07.050 31442403 10.1016/j.cell.2019.07.050 PMC7357241

[60] Boettcher M, Schacker AL, Esser M et al (2022) Markers of neutrophil activation and extracellular trap formation predict appendicitis. Surgery 171(2):312–319. 10.1016/j.surg.2021.07.010 34373106 10.1016/j.surg.2021.07.010

