# Supplementary material for: Fasting plasma methylglyoxal concentrations are associated with higher numbers of circulating intermediate and non-classical monocytes but with lower activation of intermediate monocytes: the Maastricht Study
Source: J Endocrinol Invest. 2025 Jan 23;48(5):1257–68. doi: 10.1007/s40618-025-02536-1 (PMC12049376; doi:10.1007/s40618-025-02536-1)
Supplement: Supplementary file 1 — Supplementary Material 1 [file 40618_2025_2536_MOESM1_ESM.docx]

# Supplementary data

**Table S1 Associations between plasma MGO concentrations and neutrophil activation.**

|  | **Model** | **CD11b MFI** | **CD11c MFI** | **CD16 MFI** |
| --- | --- | --- | --- | --- |
| MGO T=0 min  (n=628) | 1 | -0.02  [-0.09; 0.06] | -0.02  [-0.10; 0.06] | -0.04  [-0.12; 0.04] |
|  | 2 | -0.01  [-0.09; 0.07] | -0.01  [-0.09; 0.07] | -0.03  [-0.11; 0.05] |
|  | 3 | -0.02  [-0.10; 0.06] | -0.01  [-0.09; 0.07] | -0.03  [-0.11; 0.05] |
|  | 4 | -0.01  [-0.09; 0.07] | -0.004  [-0.09; 0.08] | -0.01  [-0.09; 0.07] |
|  | 5 | -0.01  [-0.09; 0.07] | -0.01  [-0.09; 0.08] | -0.02  [-0.10; 0.06] |
| MGO T=120 min  (n=576) | 1 | -0.06  [-0.14; 0.02] | -0.04  [-0.12; 0.04] | -0.01  [-0.09; 0.08] |
|  | 2 | -0.06  [-0.15; 0.02] | -0.03  [-0.11; 0.06] | 0.004  [-0.08; 0.09] |
|  | 3 | -0.08  [-0.16; 0.01] | -0.03  [-0.12; 0.05] | 0.01  [-0.08; 0.09] |
|  | 4 | -0.08  [-0.17; 0.02] | -0.03  [-0.13; 0.06] | 0.04  [-0.05; 0.13] |
|  | 5 | -0.09  [-0.19; 0.01] | -0.05  [-0.15; 0.05] | 0.01  [-0.09; 0.11] |

Data are presented as regression coefficients (β) and 95% CI. Plasma MGO concentrations and expression of neutrophil activation markers (CD11b, CD11c, CD16) were ln-transformed to ensure normality and then standardized. β [95% CI] represents 1-SD difference in individual activation marker expression per 1-SD increase of plasma MGO levels. Model 1: crude model; Model 2: adjusted for age and sex; Model 3: model 2 + adjusted for BMI, education status, and smoking status; Model 4: model 3 + adjusted for SBP, glucose lowering drugs, antihypertensive drugs, and lipid lowering drugs; Model 5: model 4 + adjusted for glucose metabolism status. Abbreviations: MGO, methylglyoxal; SBP, systolic blood pressure. BMI, body mass index; MFI, mean fluorescence intensity.

**Table S2 Associations between plasma MGO concentrations and classical monocyte activation.**

|  | **Model** | **CD11b MFI** | **CD11c MFI** | **CX3CR1 MFI** | **HLA-DR MFI** |
| --- | --- | --- | --- | --- | --- |
| MGO T=0 min  (n=628) | 1 | -0.06  [-0.13; 0.02] | -0.10  [-0.18; 0.02] | -0.02  [-0.10; 0.06] | -0.05  [-0.13; 0.03] |
|  | 2 | -0.05  [-0.13; 0.03] | -0.10  [-0.17; 0.02] | -0.02  [-0.10; 0.06] | -0.04  [-0.11; 0.04] |
|  | 3 | -0.05  [-0.13; 0.03] | **-0.09**  **[-0.17; -0.01]** | -0.02  [-0.10; 0.06] | -0.02  [-0.09; 0.06] |
|  | 4 | -0.06  [-0.14; 0.02] | **-0.10**  **[-0.18; -0.02]** | -0.02  [-0.10; 0.06] | -0.02  [-0.10; 0.06] |
|  | 5 | -0.06  [-0.14; 0.02] | **-0.10**  **[-0.18; -0.02]** | -0.02  [-0.10; 0.06] | -0.01  [-0.09; 0.07] |
| MGO T=120 min (n=576) | 1 | -0.02  [-0.10; 0.06] | -0.01  [-0.10; 0.07] | 0.06  [-0.03; 0.14] | -0.05  [-0.13; 0.03] |
|  | 2 | -0.01  [-0.09; 0.08] | 0.001  [-0.08; 0.09] | 0.04  [-0.04; 0.13] | -0.03  [-0.11; 0.06] |
|  | 3 | -0.02  [-0.10; 0.07] | 0.00  [-0.09; 0.09] | 0.04  [-0.05; 0.13] | 0.001  [-0.08; 0.09] |
|  | 4 | -0.04  [-0.13; 0.06] | -0.02  [-0.12; 0.07] | 0.04  [-0.05; 0.14] | -0.02  [-0.11; 0.08] |
|  | 5 | -0.05  [-0.16; 0.05] | -0.02  [-0.12; 0.08] | 0.05  [-0.05; 0.15] | 0.03  [-0.07; 0.13] |

Data are presented as regression coefficients (β) and 95% CI. Plasma MGO concentrations and expression of monocyte activation markers (CD11b, CD11c, CX3CR1, HLA-DR) were ln-transformed to ensure normality and then standardized. β [95% CI] represents 1-SD difference in individual activation marker expression per 1-SD increase of plasma MGO levels. Model 1: crude model; Model 2: adjusted for age and sex; Model 3: model 2 + adjusted for BMI, education status, and smoking status; Model 4: model 3 + adjusted for SBP, glucose lowering drugs, antihypertensive drugs, and lipid lowering drugs; Model 5: model 4 + adjusted for glucose metabolism status. Abbreviations: MGO, methylglyoxal; SBP, systolic blood pressure. BMI, body mass index; MFI, mean fluorescence intensity.

**Table S3 Associations between plasma MGO concentrations and intermediate monocyte activation.**

|  | **Model** | **CD11b MFI** | **CD11c MFI** | **CX3CR1 MFI** | **HLA-DR MFI** |
| --- | --- | --- | --- | --- | --- |
| MGO T=0 min  (n=628) | 1 | -0.03  [-0.11; 0.05] | **-0.15**  **[-0.23; -0.07]** | **-0.09**  **[-0.17; -0.01]** | -0.07  [-0.15; 0.01] |
|  | 2 | -0.02  [-0.10; 0.06] | **-0.15**  **[-0.23; -0.07]** | **-0.10**  **[-0.18; -0.03]** | -0.07  [-0.15; 0.01] |
|  | 3 | -0.02  [-0.10; 0.05] | **-0.14**  **[-0.22; -0.06]** | **-0.10**  **[-0.18; -0.03]** | -0.06  [-0.13; 0.02] |
|  | 4 | -0.04  [-0.12; 0.04] | **-0.15**  **[-0.23; -0.07]** | **-0.10**  **[-0.18; -0.02]** | -0.06  [-0.14; 0.02] |
|  | 5 | -0.04  [-0.12; 0.04] | **-0.15**  **[-0.23; -0.07]** | **-0.11**  **[-0.19; -0.03]** | -0.05  [-0.13; 0.03] |
| MGO T=120 min (n=576) | 1 | 0.05  [-0.08; 0.09] | -0.04  [-0.12; 0.05] | 0.03  [-0.05; 0.11] | -0.02  [-0.11; 0.06] |
|  | 2 | 0.03  [-0.05; 0.11] | -0.03  [-0.11; 0.06] | -0.004  [-0.09; 0.03] | -0.02  [-0.10; 0.07] |
|  | 3 | 0.01  [-0.07; 0.10] | -0.02  [-0.11; 0.07] | 0.00  [-0.08; 0.09] | 0.01  [-0.08; 0.09] |
|  | 4 | -0.004  [-0.10; 0.09] | -0.04  [-0.13; 0.06] | -0.02  [-0.11; 0.08] | -0.004  [-0.10; 0.09] |
|  | 5 | -0.03  [-0.01; 0.01] | -0.05  [-0.15; 0.06] | -0.04  [-0.14; 0.06] | 0.03  [-0.07; 0.13] |

Data are presented as regression coefficients (β) and 95% CI. Plasma MGO concentrations and expression of monocyte activation markers (CD11b, CD11c, CX3CR1, HLA-DR) were ln-transformed to ensure normality and then standardized. β [95% CI] represents 1-SD difference in individual activation marker expression per 1-SD increase of plasma MGO levels. Model 1: crude model; Model 2: adjusted for age and sex; Model 3: model 2 + adjusted for BMI, education status, and smoking status; Model 4: model 3 + adjusted for SBP, glucose lowering drugs, antihypertensive drugs, and lipid lowering drugs; Model 5: model 4 + adjusted for glucose metabolism status. Abbreviations: MGO, methylglyoxal; SBP, systolic blood pressure. BMI, body mass index; MFI, mean fluorescence intensity.

**Table S4 Associations between plasma MGO concentrations and non-classical monocyte activation.**

|  | **Model** | **CD11b MFI** | **CD11c MFI** | **CX3CR1 MFI** | **HLA-DR MFI** |
| --- | --- | --- | --- | --- | --- |
| MGO T=0 min  (n=628) | 1 | 0.07  [-0.01; 0.15] | **-0.09**  **[-0.17; -0.02]** | -0.04  [-0.12; 0.04] | -0.02  [-0.10; 0.06] |
|  | 2 | **0.09**  **[0.01; 0.16]** | **-0.08**  **[-0.16; -0.001]** | -0.05  [-0.13; 0.03] | -0.004  [-0.08; 0.08] |
|  | 3 | 0.08  [0.00; 0.16] | **-0.08**  **[-0.16; -0.01]** | -0.06  [-0.14; 0.02] | -0.01  [-0.09; 0.07] |
|  | 4 | 0.06  [-0.02; 0.14] | **-0.10**  **[-0.18; -0.02]** | -0.06  [-0.14; 0.02] | -0.01  [-0.09; 0.07] |
|  | 5 | 0.06  [-0.02; 0.14] | **-0.10**  **[-0.18; -0.01]** | -0.07  [-0.15; 0.01] | -0.01  [-0.09; 0.07] |
| MGO T=120 min (n=576) | 1 | -0.03  [-0.11; 0.05] | -0.03  [-0.11; 0.05] | 0.06  [-0.02; 0.14] | -0.03  [-0.11; 0.05] |
|  | 2 | 0.02  [-0.07; 0.10] | 0.01  [-0.08; 0.09] | 0.04  [-0.05; 0.12] | 0.01  [-0.07; 0.10] |
|  | 3 | -0.001  [-0.09; 0.08] | 0.003  [-0.08; 0.09] | 0.03  [-0.06; 0.11] | 0.01  [-0.08; 0.10] |
|  | 4 | -0.02  [-0.11; 0.07] | -0.004  [-0.10; 0.09] | 0.01  [-0.09; 0.10] | -0.003  [-0.10; 0.09] |
|  | 5 | -0.04  [-0.14; 0.06] | -0.003  [-0.10; 0.10] | -0.02  [-0.12; 0.08] | 0.003  [-0.10; 0.10] |

Data are presented as regression coefficients (β) and 95% CI. Plasma MGO concentrations and expression of monocyte activation markers (CD11b, CD11c, CX3CR1, HLA-DR) were ln-transformed to ensure normality and then standardized. β [95% CI] represents 1-SD difference in individual activation marker expression per 1-SD increase of plasma MGO levels. Model 1: crude model; Model 2: adjusted for age and sex; Model 3: model 2 + adjusted for BMI, education status, and smoking status; Model 4: model 3 + adjusted for SBP, glucose lowering drugs, antihypertensive drugs, and lipid lowering drugs; Model 5: model 4 + adjusted for glucose metabolism status. Abbreviations: MGO, methylglyoxal; SBP, systolic blood pressure. BMI, body mass index; MFI, mean fluorescence intensity.

**Table S5 Sensitivity analyses of the associations between plasma MGO levels and immune cell counts.**

|  | **Model** | **Neutrophils** | **Monocytes** | **Classical monocytes** | **Intermediate monocytes** | **Non-classical monocytes** |
| --- | --- | --- | --- | --- | --- | --- |
| **Replacement of BMI by waist circumference** | | | | | | |
| MGO T=0 min  (n=696) | 5a | 0.004  [-0.07; 0.08] | 0.03  [-0.04; 0.11] | -0.01  [-0.09; 0.06] | **0.09**  **[0.02; 0.17]** | **0.08**  **[0.002; 0.15]** |
|  | 5b | 0.001  [-0.07; 0.07] | 0.03  [-0.04; 0.10] | -0.02  [-0.09; 0.06] | **0.09**  **[0.02; 0.16]** | **0.08**  **[0.001; 0.15]** |
| MGO T=120 min  (n=639) | 5a | 0.07  [-0.02; 0.16] | 0.05  [-0.04; 0.14] | 0.04  [-0.05; 0.13] | 0.09  [-0.001; 0.18] | 0.06  [-0.03; 0.15] |
|  | 5b | 0.06  [-0.03; 0.15] | 0.05  [-0.04; 0.14] | 0.04  [-0.05; 0.13] | 0.08  [-0.01; 0.17] | 0.06  [-0.03; 0.15] |
| **Additional adjustment for DHD score and physical activity** | | | | | | |
| MGO T=0 min  (n=599) | 5a | 0.07  [-0.01; 0.14] | 0.04  [-0.04; 0.12] | 0.01  [-0.07; 0.09] | 0.06  [-0.02; 0.14] | 0.07  [-0.004; 0.02] |
|  | 5c | 0.07  [-0.01; 0.14] | 0.04  [-0.04; 0.12] | 0.01  [-0.07; 0.09] | 0.07  [-0.01; 0.15] | 0.08  [-0.002; 0.16] |
| MGO T=120 min  (n=555) | 5a | 0.02  [-0.07; 0.12] | 0.05  [-0.05; 0.15] | 0.03  [-0.06; 0.13] | 0.09  [-0.01; 0.19] | 0.07  [-0.03; 0.17] |
|  | 5c | 0.02  [-0.07; 0.12] | 0.05  [-0.05; 0.15] | 0.03  [-0.07; 0.13] | 0.09  [-0.01; 0.19] | 0.07  [-0.08; 0.17] |

Data are presented as regression coefficients (β) and 95% CI. Plasma MGO concentrations and numbers of immune cells (neutrophils, monocytes, and monocyte subsets: classical, intermediate, and non-classical monocytes) were ln-transformed to ensure normality and then standardized. β [95% CI] represents 1-SD difference in immune cell counts per 1-SD increase of plasma MGO levels.

Model 5a: adjustment for age, sex, for BMI, education status, smoking status, SBP, glucose lowering drugs, antihypertensive drugs, lipid lowering drugs, and glucose metabolism status;

Model 5b: adjustment for age, sex, for waist circumference, education status, smoking status, SBP, glucose lowering drugs, antihypertensive drugs, lipid lowering drugs, and glucose metabolism status;

Model 5c: model 5a + adjustment for DHD score and physical activity.

Abbreviations: MGO, methylglyoxal; SBP, systolic blood pressure. BMI, body mass index. DHD: Dutch healthy diet.

**Table S6 Sensitivity analyses of the associations between plasma MGO levels and immune cell activation scores.**

|  | **Model** | **Neutrophils** | **Classical monocytes** | **Intermediate monocytes** | **Non-classical monocytes** |
| --- | --- | --- | --- | --- | --- |
| **Replacement of BMI by waist circumference** | | | | | |
| MGO T=0 min  (n=628) | 5a | -0.01  [-0.10; 0.07] | -0.07  [-0.15; 0.01] | **-0.14**  **[-0.22; -0.06]** | -0.05  [-0.13; 0.03] |
|  | 5b | -0.02  [-0.10; 0.07] | -0.07  [-0.15; 0.01] | **-0.14**  **[-0.22; -0.06]** | -0.05  [-0.13; 0.03] |
| MGO T=120 min  (n=576) | 5a | -0.05  [-0.15; 0.05] | 0.003  [-0.10; 0.10] | -0.03  [-0.13; 0.07] | -0.02  [-0.12; 0.08] |
|  | 5b | -0.06  [-0.16; 0.04] | 0.00  [-0.10; 0.10] | -0.03  [-0.13; 0.07] | -0.03  [-0.13; 0.07] |
| **Additional adjustment for DHD score and physical activity** | | | | | |
| MGO T=0 min  (n=543) | 5a | -0.01  [-0.10; 0.08] | -0.02  [-0.11; 0.06] | -0.08  [-0.17; 0.01] | 0.02  [-0.06; 0.11] |
|  | 5c | -0.01  [-0.10; 0.08] | -0.02  [-0.11; 0.06] | -0.08  [-0.17; 0.01] | 0.02  [-0.06; 0.10] |
| MGO T=120 min  (n=503) | 5a | -0.04  [-0.15; 0.07] | -0.01  [-0.11; 0.10] | -0.09  [-0.20; 0.02] | -0.04  [-0.14; 0.06] |
|  | 5c | -0.04  [-0.15; 0.07] | -0.01  [-0.12; 0.10] | -0.09  [-0.20; 0.02] | -0.04  [-0.15; 0.06] |

Data are presented as β [95% CI]. Plasma MGO concentrations were ln-transformed to ensure normality and then standardized. β [95% CI] represents 1-SD difference in immune cell activation scores per 1-SD increase of plasma MGO levels.

Model 5a: adjustment for age, sex, for BMI, education status, smoking status, SBP, glucose lowering drugs, antihypertensive drugs, lipid lowering drugs, and glucose metabolism status;

Model 5b: adjustment for age, sex, for waist circumference, education status, smoking status, SBP, glucose lowering drugs, antihypertensive drugs, lipid lowering drugs, and glucose metabolism status;

Model 5c: model 5a + adjustment for DHD score and physical activity.

Abbreviations: MGO, methylglyoxal; SBP, systolic blood pressure. BMI, body mass index. DHD: Dutch healthy diet.
