# Supplementary material for: Fasting plasma methylglyoxal concentrations are associated with higher numbers of circulating intermediate and non-classical monocytes but with lower activation of intermediate monocytes: the Maastricht Study
Source: J Endocrinol Invest. 2025 Jan 23;48(5):1257–68. doi: 10.1007/s40618-025-02536-1 (PMC12049376; doi:10.1007/s40618-025-02536-1)
Supplement: Supplementary file 2 — Supplementary Material 2 [file 40618_2025_2536_MOESM2_ESM.docx]

**Additional Tables for rebuttal**

**Table A1 Associations between immune cell counts and CVD history (population with fasting MGO)**

|  | **Model** | **Neutrophils** | **Monocytes** | **Classical monocytes** | **Intermediate monocytes** | **Non-classical monocytes** |
| --- | --- | --- | --- | --- | --- | --- |
| CVD  (n=695) | 1 | **1.42**  **[1.13; 1.79]** | **1.33**  **[1.08; 1.63]** | **1.26**  **[1.03; 1.56]** | **1.33**  **[1.08; 1.64]** | **1.27**  **[1.03; 1.56]** |
|  | 2 | **1.37**  **[1.08; 1.75]** | 1.23  [0.98; 1.53] | 1.17  [0.94; 1.46] | 1.21  [0.97; 1.51] | 1.19  [0.95; 1.48] |
|  | 3 | 1.28  [0.99; 1.67] | 1.16  [0.93; 1.46] | 1.11  [0.88; 1.40] | 1.17  [0.93; 1.46] | 1.18  [0.95; 1.48] |
|  | 4 | 1.15  [0.89; 1.49] | 1.09  [0.87; 1.38] | 1.05  [0.83; 1.32] | 1.11  [0.88; 1.40] | 1.16  [0.93; 1.46] |
|  | 5 | 1.15  [0.89; 1.50] | 1.10  [0.87; 1.38] | 1.05  [0.83; 1.33] | 1.11  [0.88; 1.41] | 1.16  [0.93; 1.46] |

Data are analyzed by logistic regression and presented as odds ratios (OR) and 95% CI. OR [95% CI] represents per standard deviation (SD) increase of independent variable (immune cell counts). Numbers of immune cells were ln-transformed to ensure normality and then standardized. Model 1: crude model; Model 2: adjusted for age and sex; Model 3: model 2 + adjusted for body mass index, education status, and smoking status; Model 4: model 3 + adjusted for systolic blood pressure, glucose lowering drugs, antihypertensive drugs, lipid lowering drugs; Model 5: model 4 + adjusted for glucose metabolism status.

**Table A2 Associations between immune cell activation scores and CVD history (population with fasting MGO)**

|  | **Model** | **Neutrophils** | **Classical**  **monocytes** | **Intermediate monocytes** | **Non-classical monocytes** |
| --- | --- | --- | --- | --- | --- |
| CVD  (n=627) | 1 | 0.81  [0.66; 1.00] | 0.92  [0.75; 1.12] | 1.07  [0.83; 1.37] | 1.12  [0.89; 1.40] |
|  | 2 | 0.81  [0.65; 1.01] | 0.93  [0.74; 1.17] | 1.12  [0.82; 1.51] | 1.21  [0.94; 1.56] |
|  | 3 | 0.81  [0.65; 1.01] | 0.97  [0.76; 1.22] | 1.15  [0.84; 1.57] | 1.18  [0.91; 1.53] |
|  | 4 | 0.85  [0.68; 1.07] | 0.94  [0.75; 1.18] | 1.11  [0.82; 1.51] | 1.11  [0.86; 1.44] |
|  | 5 | 0.85  [0.68; 1.07] | 0.94  [0.75; 1.19] | 1.11  [0.82; 1.52] | 1.12  [0.86; 1.45] |

Data are analyzed by logistic regression and presented as odds ratios (OR) and 95% CI. OR [95% CI] represents per standard deviation (SD) increase of independent variable (immune cell activation scores). Model 1: crude model; Model 2: adjusted for age and sex; Model 3: model 2 + adjusted for body mass index, education status, and smoking status; Model 4: model 3 + adjusted for systolic blood pressure, glucose lowering drugs, antihypertensive drugs, lipid lowering drugs; Model 5: model 4 + adjusted for glucose metabolism status.

**Table A3 Associations between immune cell counts and CVD history**

|  | **Model** | **Neutrophils** | **Monocytes** | **Classical monocytes** | **Intermediate monocytes** | **Non-classical monocytes** |
| --- | --- | --- | --- | --- | --- | --- |
| CVD  (n=1059) | 1 | **1.35**  **[1.14; 1.60]** | **1.38**  **[1.17; 1.62]** | **1.33**  **[1.13; 1.57]** | **1.30**  **[1.11; 1.52]** | **1.28**  **[1.09; 1.51]** |
|  | 2 | **1.32**  **[1.10; 1.58]** | **1.26**  **[1.06; 1.50]** | **1.22**  **[1.03; 1.45]** | **1.18**  **[1.00; 1.40]** | **1.20**  **[1.01; 1.42]** |
|  | 3 | 1.20  [0.99; 1.44] | **1.19**  **[1.00; 1.42]** | 1.15  [0.96; 1.37] | 1.12  [0.95; 1.33] | **1.23**  **[1.03; 1.47]** |
|  | 4 | 1.06  [0.88; 1.28] | 1.12  [0.93; 1.33] | 1.08  [0.91; 1.30] | 1.06  [0.89; 1.27] | 1.18  [0.98; 1.41] |
|  | 5 | 1.07  [0.88; 1.29] | 1.12  [0.94; 1.34] | 1.09  [0.91; 1.30] | 1.07  [0.89; 1.27] | 1.18  [0.98; 1.42] |

Data are analyzed by logistic regression and presented as odds ratios (OR) and 95% CI. OR [95% CI] represents per standard deviation (SD) increase of independent variable (immune cell counts). Numbers of immune cells were ln-transformed to ensure normality and then standardized. Model 1: crude model; Model 2: adjusted for age and sex; Model 3: model 2 + adjusted for body mass index, education status, and smoking status; Model 4: model 3 + adjusted for systolic blood pressure, glucose lowering drugs, antihypertensive drugs, lipid lowering drugs; Model 5: model 4 + adjusted for glucose metabolism status.

**Table A4 Associations between immune cell activation scores and CVD history**

|  | **Model** | **Neutrophils** | **Classical**  **monocytes** | **Intermediate monocytes** | **Non-classical monocytes** |
| --- | --- | --- | --- | --- | --- |
| CVD  (n=987) | 1 | **0.84**  **[0.72; 0.99]** | 0.98  [0.83; 1.15] | 1.06  [0.88; 1.27] | 1.10  [0.93; 1.30] |
|  | 2 | 0.85  [0.72; 1.01] | 1.00  [0.84; 1.20] | 1.11  [0.90; 1.36] | 1.16  [0.96; 1.38] |
|  | 3 | 0.85  [0.72; 1.01] | 1.03  [0.86; 1.23] | 1.12  [0.91; 1.38] | 1.11  [0.92; 1.33] |
|  | 4 | 0.88  [0.74; 1.05] | 1.00  [0.84; 1.20] | 1.07  [0.87; 1.32] | 1.05  [0.87; 1.26] |
|  | 5 | 0.89  [0.74; 1.05] | 1.00  [0.84; 1.20] | 1.07  [0.87; 1.32] | 1.05  [0.88; 1.26] |

Data are analyzed by logistic regression and presented as odds ratios (OR) and 95% CI. OR [95% CI] represents per standard deviation (SD) increase of independent variable (immune cell activation scores). Model 1: crude model; Model 2: adjusted for age and sex; Model 3: model 2 + adjusted for body mass index, education status, and smoking status; Model 4: model 3 + adjusted for systolic blood pressure, glucose lowering drugs, antihypertensive drugs, lipid lowering drugs; Model 5: model 4 + adjusted for glucose metabolism status.

**Table A5 Associations between immune cell counts and CVD history, stratified for glucose metabolism status**

|  | **Model** | **Neutrophils** | **Monocytes** | **Classical monocytes** | **Intermediate monocytes** | **Non-classical monocytes** |
| --- | --- | --- | --- | --- | --- | --- |
| **CVD (total)** | | | | | | |
| NGM  (n=577) | 1 | 1.05  [0.81; 1.36] | **1.29**  **[1.00; 1.65]** | 1.23  [0.95; 1.59] | 1.20  [0.94; 1.54] | **1.34**  **[1.03; 1.76]** |
|  | 2 | 1.12  [0.84; 1.51] | 1.28  [0.98; 1.67] | 1.22  [0.94; 1.60] | 1.15  [0.88; 1.49] | 1.31  [0.98; 1.76] |
|  | 3 | 1.12  [0.83; 1.53] | 1.27  [0.97; 1.67] | 1.23  [0.93; 1.62] | 1.12  [0.85; 1.46] | 1.31  [0.97; 1.78] |
|  | 4 | 1.07  [0.80; 1.45] | 1.27  [0.96; 1.68] | 1.22  [0.92; 1.62] | 1.15  [0.86; 1.52] | 1.32  [0.97; 1.81] |
| Prediabetes  (n=142) | 1 | 1.20  [0.79; 1.73] | 1.04  [0.68; 1.59] | 1.04  [0.68; 1.60] | 0.98  [0.64; 1.50] | 0.91  [0.60; 1.40] |
|  | 2 | 1.19  [0.77; 1.86] | 0.89  [0.57; 1.40] | 0.89  [0.57; 1.40] | 0.91  [0.58; 1.42] | 0.89  [0.57; 1.40] |
|  | 3 | 1.03  [0.64; 1.67] | 0.85  [0.54; 1.35] | 0.81  [0.51; 1.29] | 0.94  [0.58; 1.50] | 1.11  [0.68; 1.81] |
|  | 4 | 0.87  [0.51; 1.48] | 0.70  [0.42; 1.17] | 0.66  [0.39; 1.12] | 0.80  [0.46; 1.39] | 0.99  [0.59; 1.68] |
| T2D  (n=340) | 1 | **1.29**  **[1.01; 1.65]** | **1.32**  **[1.03; 1.68]** | **1.28**  **[1.01; 1.64]** | 1.22  [0.96; 1.55] | 1.20  [0.94; 1.53] |
|  | 2 | **1.30**  **[1.01; 1.68]** | 1.26  [0.98; 1.62] | 1.23  [0.96; 1.59] | 1.18  [0.92; 1.50] | 1.15  [0.90; 1.48] |
|  | 3 | 1.25  [0.96; 1.63] | 1.22  [0.94; 1.57] | 1.18  [0.91; 1.53] | 1.15  [0.90; 1.47] | 1.17  [0.91; 1.52] |
|  | 4 | 1.17  [0.89; 1.53] | 1.15  [0.88; 1.50] | 1.13  [0.86; 1.48] | 1.10  [0.86; 1.42] | 1.11  [0.85; 1.45] |

Data are analyzed by logistic regression and presented as odds ratios (OR) and 95% CI. OR [95% CI] represents per standard deviation (SD) increase of independent variable (immune cell counts). Numbers of immune cells were ln-transformed to ensure normality and then standardized. Model 1: crude model; Model 2: adjusted for age and sex; Model 3: model 2 + adjusted for body mass index, education status, and smoking status; Model 4: model 3 + adjusted for systolic blood pressure, glucose lowering drugs, antihypertensive drugs, lipid lowering drugs.

**Table A6 Associations between MGO concentrations and CVD history**

|  | **Model** | **MGOt0**  **(n=695)** | **MGOt0**  **(n=3183)** | **MGOt120**  **(n=638)** | **MGOt120**  **(n=2929)** |
| --- | --- | --- | --- | --- | --- |
| CVD | 1 | 1.06  [0.87; 1.30] | **1.16**  **[1.06; 1.27]** | 1.19  [0.97; 1.46] | **1.22**  **[1.11; 1.35]** |
|  | 2 | 0.99  [0.81; 1.23] | **1.10**  **[1.01; 1.21]** | 1.07  [0.86; 1.33] | **1.12**  **[1.01; 1.24]** |
|  | 3 | 0.96  [0.77; 1.19] | 1.06  [0.96; 1.16] | 1.01  [0.80; 1.27] | 1.06  [0.96; 1.18] |
|  | 4 | 0.88  [0.70; 1.11] | 0.98  [0.88; 1.09] | 0.87  [0.67; 1.14] | 0.96  [0.85; 1.09] |
|  | 5 | 0.89  [0.71; 1.12] | 0.99  [0.89; 1.10] | 0.85  [0.64; 1.13] | 0.98  [0.86; 1.11] |

Data are analyzed by logistic regression and presented as odds ratios (OR) and 95% CI. OR [95% CI] represents per standard deviation (SD) increase of independent variable (MGO concentrations). Model 1: crude model; Model 2: adjusted for age and sex; Model 3: model 2 + adjusted for body mass index, education status, and smoking status; Model 4: model 3 + adjusted for systolic blood pressure, glucose lowering drugs, antihypertensive drugs, lipid lowering drugs; Model 5: model 4 + adjusted for glucose metabolism status.
